# Exploring the impact of urogenital organ displacement after abdominoperineal resection on urinary and sexual function

**DOI:** 10.1007/s00384-022-04234-3

**Published:** 2022-08-31

**Authors:** Sarah Sharabiany, Saskia I. Kreisel, Gaby J. Strijk, Robin D. Blok, Judith Bosschieter, Ellen T. M. Laan, Christopher Cunningham, Roel Hompes, Gijsbert D. Musters, Pieter J. Tanis

**Affiliations:** 1 Department of Surgery, Amsterdam UMC, University of Amsterdam, Amsterdam, the Netherlands; 2 Department of Urology, Amsterdam UMC, VU University, Amsterdam, the Netherlands; 3 Department of Sexology, Amsterdam UMC, University of Amsterdam, Amsterdam, the Netherlands; 4 Department of Colorectal Surgery, Churchill Hospital, Oxford University Hospitals NHS Foundation Trust, Oxford, UK; 5 Department of Surgery, Amsterdam UMC, University of Amsterdam, Cancer Centre Amsterdam, Amsterdam, the Netherlands; 6 Department of Surgical Oncology and Gastrointestinal Surgery, Erasmus MC, Doctor Molewaterplein 30, 3015 GD Rotterdam, the Netherlands

**Keywords:** Abdominoperineal resection, Urinary, Sexual, Function

## Abstract

**Purpose:**

This study aimed to establish the functional impact of displacement of urogenital organs after abdominoperineal resection (APR) using validated questionnaires.

**Methods:**

Patients who underwent APR for primary or recurrent rectal cancer (2001–2018) with evaluable pre- and postoperative radiological imaging and completed urinary (UDI-6, IIQ-7) and sexual questionnaires (male, IIEF; female, FSFI, FSDS-R) were included from 16 centers. Absolute displacement of the internal urethral orifice, posterior bladder wall, distal end of the prostatic urethra, and cervix were correlated to urogenital function by calculating Spearman’s Rho (*ρ*). Median function scores were compared between minimal or substantial displacement using median split.

**Results:**

There were 89 male and 36 female patients included, of whom 45 and 19 were sexually active after surgery. Absolute displacement of the internal urethral orifice and posterior bladder wall was not correlated with UDI-6 in men (*ρ* = 0.119 and *ρ* = 0.022) nor in women (*ρ* =  − 0.098 and *ρ* =  − 0.154). In men with minimal and substantial displacement of the internal urethral orifice, median UDI-6 scores were 10 (IQR 0–22) and 17 (IQR 5–21), respectively, with corresponding scores of 25 (IQR 10–46) and 21 (IQR 16–36) in women. Displacement of the cervix and FSDS-R were correlated (*ρ* = 0.433) in sexually active patients.

**Conclusion:**

This first analysis on functional impact of urogenital organ displacement after APR suggests that more displacement of the cervix might be associated with worse sexual function, while the data does not indicate any potential functional impact of bladder displacement. Studies are needed to further explore this underexposed topic.

**Supplementary Information:**

The online version contains supplementary material available at 10.1007/s00384-022-04234-3.

## Introduction

Urogenital function is commonly affected after abdominoperineal resection (APR) in both men and women [1]. Factors known to be associated with impaired urogenital function after APR and rectal cancer treatment in general include preoperative radiotherapy, direct surgical autonomic nerve injury, and pre-existing comorbidities [2]. Some patients present with severe urinary and/or sexual dysfunction and indicate themselves that this might be related to postoperative anatomical changes. Female patients in particular will describe maneuvres to assist emptying their bladder that has prolapsed onto the closed perineal skin, for example, by giving manual compression behind the introitus. Regarding sexual dysfunction, women can report that sexual intercourse was no longer possible after APR due to kinking of the vagina. These complaints might also be associated with the presence of a symptomatic perineal hernia. Therefore, it has been our hypothesis that urogenital dysfunction after non-restorative rectal cancer resection can have a mechanical component among all other etiological factors.

In a previous study, we quantified that the creation of a large empty cavity in the pelvis after APR can result in substantial displacement of the urogenital organs, and high inter-individual variability was observed [3]. The degree of these anatomical changes may theoretically affect the function of the organs in various ways. Dorsal displacement of the bladder in both genders can result in bladder emptying difficulties and risk of overflow incontinence. Analyzing numerous postoperative pelvic images in our previous study revealed several severe anatomical disturbances of the internal genital organs, varying from slight angulation of the vagina to complete prolapse of the top of the vagina through the pelvic floor defect. In contrast to what we expected, also substantial displacement of the prostate was observed.

Multiple factors contribute to the pathophysiology of urinary and sexual dysfunction after APR for rectal cancer. The main focus has been on radiotherapy and surgery-induced autonomic nerve damage, but it has not yet been investigated if there is an association between postoperative displacement of urogenital organs and urogenital function. We hypothesized that the magnitude of urogenital organ displacement contributes to the severity of urogenital dysfunction. If a correlation can be demonstrated, this can then be further analyzed to determine the independent role in functional impairments besides all other risk factors. The aim of this multicenter cohort study was to determine whether any potential correlation between urogenital organ displacement and bladder or sexual function can be established using validated questionnaires in male and female patients who have undergone APR for rectal cancer.

## Materials and methods

### Patients

In this study, three existing databases consisting of patients who underwent APR for primary or recurrent rectal cancer between 2001 and 2018 were used [4–6]. Patients were included if pre- and postoperative pelvic imaging as well as completed urogenital questionnaires postoperatively were available. Patients with total exenteration were excluded.

Postoperative pelvic imaging was performed according to routine daily practice in the cohort series or as part of the randomized controlled BIOPEX trial. Urogenital questionnaires were sent to all patients postoperatively. Part of the questionnaires was also used in the long-term follow-up of the BIOPEX trial [7]. Patients who participated in the BIOPEX trial already completed quality of life questionnaires preoperatively. This trial had been approved by the ethical review board of the Academic Medical Center, and informed consent was obtained [6]. The need for informed consent was waived for the retrospective studies [4, 5].

### Quantification of urogenital organ displacement

Urogenital organ displacement was measured in the midsagittal plane of pelvic imaging. Reference lines between fixed bony structures were used as a coordinate system, which resulted in pre- and postoperative X and Y coordinates of the anatomical landmarks (Supplementary Figure S1). Three anatomical landmarks were determined for both genders. The two common landmarks were the internal urethral orifice and the posterior bladder wall. In male, the third anatomical landmark was the distal end of the prostatic urethra and in female the cervix (or top of the vagina in case of hysterectomy).

A more detailed description of the reference lines, anatomical landmarks, and calculation of displacement has been published previously [3].

Measurements were performed by three investigators (SSH, SIK, or GJS). A fourth investigator (PJT) was consulted in case of discrepancies to reach consensus through discussion.

### Questionnaires

The European Organization for Research and Treatment for Cancer Quality of Life Questionnaire Colorectal Cancer (EORTC QLQ-CR29) was used to obtain information regarding the baseline urogenital function [8]. The symptom scale (frequency, incontinence, dysuria, and impotence/dyspareunia) ranges from 0 to 100, with a higher score representing more symptoms. The functional scale (sexual interest) ranges from 0 to 100, with a higher scale score representing a better level of functioning. No cut-off values were available.

Postoperatively, men and women completed validated questionnaires regarding urinary and sexual function (Supplementary Tables S1–S5). Two questionnaires were used to examine urinary function for both genders. The Urogenital Distress Inventory consists of six questions (UDI-6) with outcomes between zero and 75 [9]. This questionnaire differentiates between irritative, stress, and obstructive urinary dysfunction. Due to the anatomical direction of displacement and thereby assumed obstructive dysfunction, this domain has been examined separately. Furthermore, the Incontinence Impact Questionnaire includes seven questions (IIQ-7) which are divided into four domains, namely physical activity, travel, social contacts, and emotional wellbeing [9]. Minimum score is zero, and maximum is 100. For both questionnaires, higher scores implicate more complaints. The minimal clinically important difference (MCID) is 11 and 16 points for the UDI-6 and IIQ-7, respectively [10]. The MCID represents the smallest change in score associated with a clinically meaningful change in a questionnaire. This is essential to interpret questionnaire results when assessing between-group differences.

To examine sexual function in male patients, the International Index for Erectile Function (IIEF) was used [11]. This questionnaire consists of 15 questions with outcomes between zero and 30, whereby scores < 14 are associated with impaired sexual function. Male patients were scored as sexually inactive and having no intercourse if patients answered ‘’no sexual activity’’ to questions 1 and/or 2 and ‘’no intercourse’’ to questions 6, 7, and/or 8, respectively.

Female sexual function was assessed using two questionnaires. The Female Sexual Function Index (FSFI) questionnaire contains 19 questions with an outcome between two and 36, where a score of < 26.55 is seen as dysfunction [12]. This questionnaire covers the domains: desire, arousal, lubrication, orgasm, satisfaction, and pain. We hypothesized that displacement of the cervix could be associated with pain complaints, and therefore we examined this domain separately. Female patients were scored as sexually inactive and having no intercourse if patients answered ‘’no sexual activity’’ to the domains arousal, lubrication, and/or orgasm and ‘’no intercourse’’ to questions 17, 18, and/or 19, respectively. The MCID for this questionnaire is only determined per domain, which is 0.1 point for the pain domain. Furthermore, the Female Sexual Distress Scale-Revised (FSDS-R) questionnaire was used, consisting of 13 questions with an outcome between zero and 52. An outcome of > 11 points reflects sexual dysfunction. The MCID, to interpret between-group differences, is 6 for this questionnaire.

### Outcome

The primary outcome was the degree of urinary and sexual dysfunction in male and female patients after urogenital organ displacement following APR for rectal cancer.

### Statistical analysis

Categorical data were compared using the chi-square test and numerical data with the independent *t* test or Mann–Whitney *U* (MWU) test according to distribution.

The questionnaires were analyzed according to the manuals, whereas total scores were only calculated if all domains were completed. Analysis was based on radiological imaging at the nearest date to the day the questionnaire was completed. UDI-6 and IIQ-7 were correlated to the absolute displacement of the internal urethral orifice and posterior bladder wall for both genders. IIEF was correlated with the absolute displacement of the distal end of the prostatic urethra. FSFI total score, FSFI pain domain, and FSDS-R were correlated with absolute displacement of the cervix. All the above analyses were repeated with rotation of the different anatomical landmarks [3]. The correlation between the displacement and questionnaires were analyzed with the Spearman’s rank correlation coefficient and were graphically displayed in scatterplots.

Patients were divided into sexually active and inactive for baseline characteristics and for correlation analyses between displacement and sexual function. Patients not having intercourse were excluded from the analyses regarding FSFI domain pain.

Subgroup analyses were performed by categorizing patients into minimal or substantial displacement using median split. Functional scores for subgroups were analyzed using the MWU-test and presented as median with interquartile range (IQR). The MCID was taken into account to interpret observed differences.

The statistical significance level was set at a *P* value of < 0.05. Statistical analysis was performed using SPSS software for Windows version 26 (IBM Corp, Armonk, NY).

## Results

### Patient characteristics

**Fig. 1:**
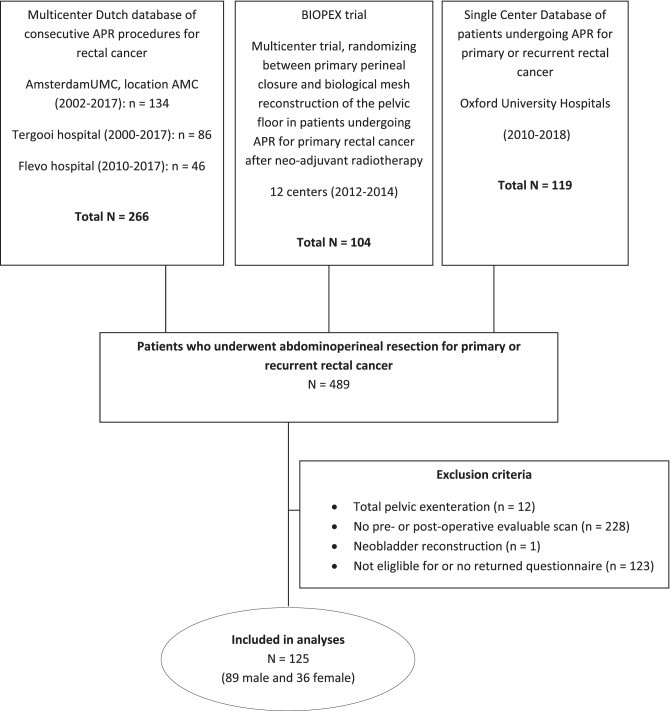
Flow diagram showing initial number of participants and those excluded for any reason

A total of 489 patients underwent APR for rectal cancer in fourteen centers in the Netherlands (2001–2018) and two centers in the UK (2010–2018). After exclusion of total pelvic exenteration (*n* = 12), neobladder reconstruction (*n* = 1), no pre- or postoperative evaluable scan (*n* = 228), and not eligible for or no returned questionnaire (*n* = 123), a total of 125 patients were included (Fig. 1). Mean age at surgery was 64 years ± 12.2, and 89 (71%) were male. Male patients who were sexually active were younger (59 ± 12 vs. 69 ± 9; *P* < 0.001). Prior surgery of the lower urinary tract was performed in two male patients, consisting of partial bladder resection and transurethral resection of the prostate. Almost one-third of the female patients (28%) had undergone surgery of their reproductive system, existing of hysterectomy (*n* = 4), tubular sterilization (*n* = 3), ovarian cystectomy (*n* = 1), oophorectomy (*n* = 1), and cesarean section (*n* = 1). Neoadjuvant radiotherapy was given in 111 patients (92%). Multivisceral resection was performed in 32 male (36%) and 17 female (47%) patients, and sexually inactive male patients had more often multivisceral resection (41% (17/42) vs. 31% (14/45); *P* = 0.381). Two women underwent hysterectomy as part of the APR (7%). Sexually active female patients had more often an omentoplasty (53% (10/19) vs. 39% (5/13); *P* = 0.491) and less often biological mesh closure of the perineum (32% (6/19) vs. 62% (8/13); *P* = 0.149) (Table 1). The median time between APR and day the questionnaire was completed was 50 months (IQR 45–75), and between most recent imaging and completed questionnaire 22 months (IQR 5–43).

**Table 1:** Baseline characteristics

		**Male total (*n* = 89)**	**Female total (*n* = 36)**	**Male sexually active (*n* = 45)**	**Male sexually inactive (*n* = 42)**	**Female sexually active (*n* = 19)**	**Female sexually inactive (*n* = 13)**
Age	Years (mean ± SD)	64 ± 12	64 ± 14	59 ± 12	69 ± 9	62 ± 12	63 ± 18
BMI	Kg/m^2^ (mean ± SD)	27 ± 4	27 ± 5	27 ± 4	27 ± 3	27 ± 6	26 ± 4
Prior pelvic surgery	Total*	7/89 (8)	13/36 (36)	4/45 (9)	3/42 (7)	8/19 (42)	5/13 (39)
	Female reproductive system^#^	-	10/36 (28)	-	-	7/19 (37)	3/13 (23)
	Male reproductive system^&^	1/89 (1)	-	0/45 (0)	1/42 (2)	-	-
	Lower urinary tract^+^	1/89 (1)	0/36 (0)	0/45 (0)	1/42 (2)	0/19 (0)	0/13 (0)
	Gastro intestinal ~	5/89 (6)	3/36 (8)	4/45 (9)	1/42 (2)	1/19 (5)	2/13 (15)
APR indication	Primary rectal cancer	86/89 (97)	35/36 (97)	42/45 (93)	42/42 (100)	18/19 (95)	13/13 (100)
	Recurrent rectal cancer	3/89 (3)	1/36 (3)	3/45 (7)	0/42 (0)	1/19 (5)	0/13 (0)
Neo-adjuvant treatment	None	9/89 (10)	2/36 (6)	5/45 (11)	4/42 (10)	1/19 (5)	1/13 (8)
	Short-course radiotherapy	13/89 (15)	8/36 (22)	7/45 (16)	6/42 (14)	4/19 (21)	3/13 (23)
	Long-course radiotherapy	14/89 (16)	11/36 (31)	8/45 (18)	6/42 (14)	5/19 (26)	4/13 (31)
	Chemoradiotherapy	53/89 (60)	15/36 (42)	25/45 (56)	26/42 (62)	9/19 (47)	5/13 (39)
APR type	Intersphincteric	6/88 (7)	0/36 (0)	2/45 (4)	4/41 (10)	0/19 (0)	0/13 (0)
	Conventional	14/88 (16)	8/36 (22)	9/45 (20)	5/41 (12)	4/19 (21)	2/13 (15)
	Extralevator	68/88 (77)	28/36 (78)	34/45 (76)	32/41 (78)	15/19 (79)	11/13 (85)
Multivisceral resection	Total	32/89 (36)	17/36 (47)	14/45 (31)	17/42 (41)	12/19 (63)	3/13 (23)
	Vaginal wall	-	9/36 (25)	-	-	7/19 (37)	1/13 (8)
	Adnex	-	3/36 (8)	-	-	2/19 (11)	1/13 (8)
	Uterus^@^	-	2/30 (7)	-	-	1/16 (6)	0/10 (0)
	Seminal vesicle	6/89 (7)	-	6/45 (13)	0/42 (0)	-	-
	(Partial) prostate	8/89 (9)	-	5/45 (11)	2/42 (5)	-	-
	(Partial) bladder	1/89 (1)	0/36 (0)	0/45 (0)	1/42 (2)	0/19 (0)	0/13 (0)
	Coccyx	18/89 (20)	3/36 (8)	4/45 (9)	14/42 (33)	2/19 (11)	0/13 (0)
	Pelvic side wall	2/89 (2)	2/36 (6)	2/45 (4)	0/42 (0)	1/19 (5)	1/13 (8)
	Presacral fascia	0/89 (0)	1/36 (3)	0/45 (0)	0/42 (0)	1/19 (5)	0/13 (0)
Omentoplasty	Total	43/89 (48)	17/36 (47)	20/45 (44)	22/42 (52)	10/19 (53)	5/13 (39)
Retroflexion uterus^+^	Total	-	1/18 (6)	-	-	1/13 (8)	0/3 (0)
Perineal closure	Primary closure	29/89 (33)	14/36 (39)	13/45 (29)	16/42 (38)	8/19 (42)	4/13 (31)
	Biological mesh	44/89 (49)	15/36 (42)	20/45 (44)	23/42 (55)	6/19 (32)	8/13 (62)
	Resorbable synthetic mesh	13/89 (15)	4/36 (11)	9/45 (20)	3/42 (7)	4/19 (21)	0/13 (0)
	Gluteal turnover flap	2/89 (2)	1/36 (3)	2/45 (4)	0/42 (0)	0/19 (0)	1/13 (8)
	Muscle flap	1/89(1)	2/36 (6)	1/45 (2)	0/42 (0)	1/19 (5)	0/13 (0)

Data are presented as absolute numbers (proportions), unless otherwise stated

*BMI* body mass index, *SD* standard deviation, *TURP* transurethral resection of the prostate, *TURBT* transurethral resection of bladder tumor, *TEM* transanal endoscopic microsurgery, *APR* abdominoperineal resection

^a^More than one of the variables below might be applicable to the same patient

^b^Female reproductive system includes sacral colpopexy, sectio caesarea, oophorectomy, hysterectomy, sterilization, excision ovarian cyst

^c^Male reproductive system includes (partial) prostatectomy and TURP

^d^Lower urinary tract includes TURB

^e^Gastrointestinal includes TEM, LAR, and perianal abscess

^f^Patients with a prior hysterectomy were excluded

^g^Patients with a prior hysterectomy or hysterectomy during operation were excluded

A comparison of baseline characteristics between patients who did and who did not return questionnaires is presented in Supplementary Table S6.

### Absolute displacement

#### Urinary function

Before APR, 30% (27/89) of male patients completed the EORTC QLQ-CR29 questionnaire. Mean score of the domain urinary frequency was 32.1 ± 24.9, of urinary incontinence 2.4 ± 8.9 and of dysuria 3.7 ± 10.7 (Supplementary Table S7). This questionnaire was completed in 25% (9/36) of female patients, and corresponding mean scores were 22.2 ± 27.6, 11.1 ± 16.7, and 0.0 ± 0.0.

The absolute displacement of the internal urethral orifice was not correlated with UDI-6 in both genders (*ρ* = 0.119, *P* = 0.285 in men vs. *ρ* =  − 0.098, *P* = 0.601 in women), even when examining the domain obstructive only (*ρ* = -0.019, *P* = 0.861 in men vs. *ρ* = 0.031, *P* = 0.870 in women). Similarly, the IIQ-7 was not correlated with this landmark in men nor in women (*ρ* = 0.149, *P* = 0.175 vs. *ρ* = -0.099, *P* = 0.602, respectively).

**Fig. 2:**
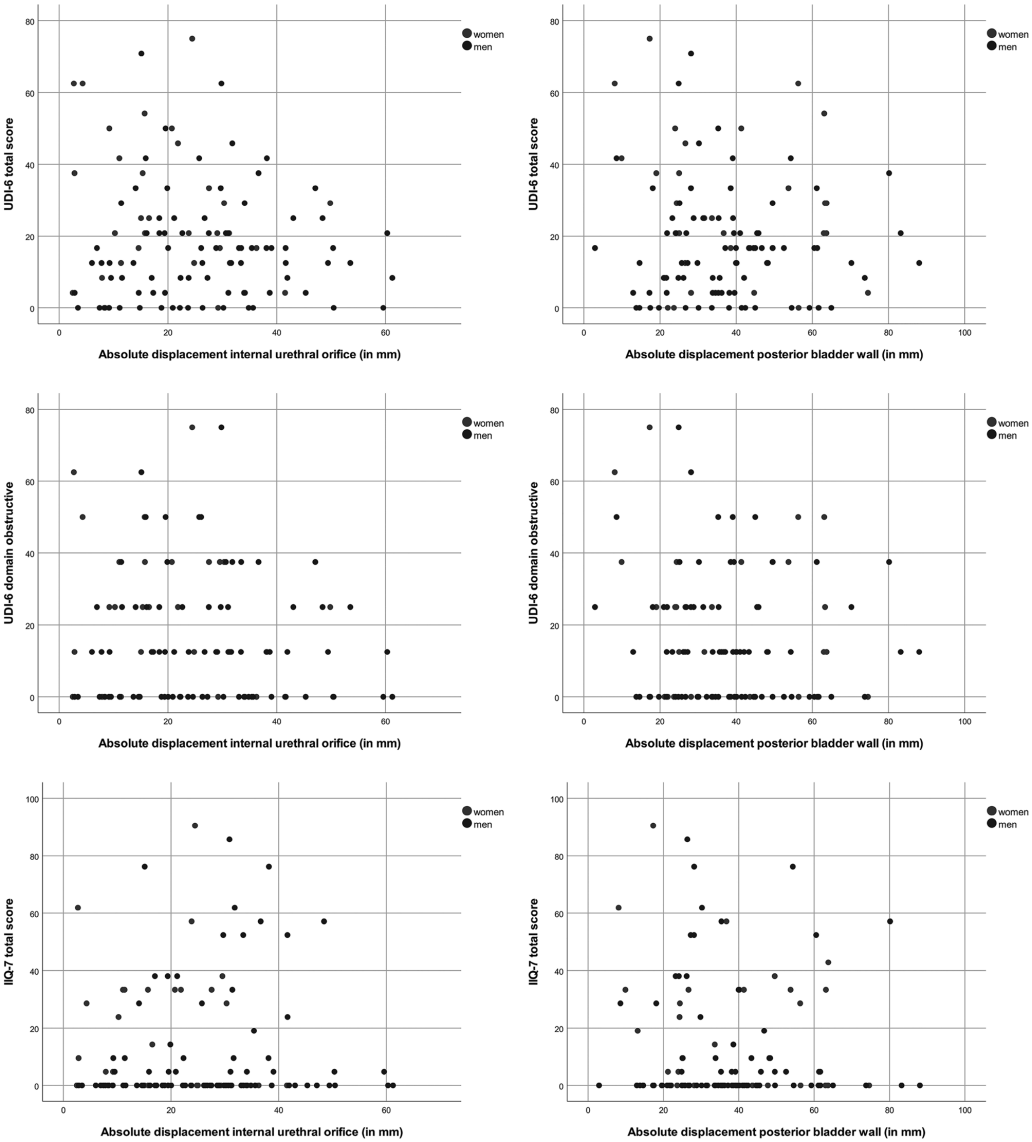
Scatterplots. Correlation of the absolute displacement in millimeters of the internal urethral orifice and posterior bladder wall with UDI-6 total score, UDI-6 domain pain, and IIQ-7 total score

Considering the posterior bladder wall, no correlation with UDI-6 was found in both genders (*ρ* = 0.022, *P* = 0.844 in men vs. *ρ* =  − 0.154, *P* = 0.391 in women). Regarding the domain obstructive of UDI-6, no correlation was found (*ρ* =  − 0.065, *P* = 0.562 in men vs. *ρ* =  − 0.170, *P* = 0.343 in women). Similarly, this landmark was not correlated to IIQ-7 (*ρ* = 0.032, *P* = 0.769 in men vs. *ρ* =  − 0.142, *P* = 0.432 in women). The Spearman’s Rho analyses are presented in Table 2, and the scatterplots are illustrated in Fig. 2a–f.

**Table 2:** Spearman correlation between the absolute displacement of the landmark and functional score

**Gender**	**Landmark**	**Questionnaire**		**Total**			**Sexually active**			**Sexually inactive**		
			**Domain**	***n***	***ρ***	***P*-value**	***n***	***ρ***	***P*-value**	***n***	***ρ***	***P*-value**
♂	Internal urethra orifice	UDI-6		82	0.119	0.285						
♂	Internal urethra orifice	UDI-6	Obstructive	83	− 0.019	0.861						
♂	Internal urethra orifice	IIQ-7		84	0.149	0.175						
♀	Internal urethra orifice	UDI-6		31	− 0.098	0.601						
♀	Internal urethra orifice	UDI-6	Obstructive	31	0.031	0.870						
♀	Internal urethra orifice	IIQ-7		30	− 0.099	0.602			Not applicable			
♂	Posterior bladder wall	UDI-6		82	0.022	0.844						
♂	Posterior bladder wall	UDI-6	Obstructive	83	− 0.065	0.562						
♂	Posterior bladder wall	IIQ-7		84	0.032	0.769						
♀	Posterior bladder wall	UDI-6		33	− 0.154	0.391						
♀	Posterior bladder wall	UDI-6	Obstructive	33	− 0.170	0.343						
♀	Posterior bladder wall	IIQ-7		33	− 0.142	0.432						
♂	Distal end of prostatic urethra	IIEF		63	0.023	0.855	36	0.015	0.929	27	− 0.253	0.204
♀	Cervix/top of vagina	FSFI		19	0.321	0.180	12	0.224	0.484	7	0.607	0.148
♀	Cervix/top of vagina	FSFI	Pain	n.a	n.a	n.a	12	0.390	0.210	n.a	n.a	n.a
♀	Cervix/top of vagina	FSDS-R		25	0.179	0.391	15	0.433	0.107	9	− 0.287	0.454

*UDI-6* Urogenital Distress Inventory short form, *IIQ-7* Incontinence Impact Questionnaire short form, *IIEF* International Index of Erectile Function, *FSFI* Female Sexual Function Index (converted), *FSDS-R* Female Sexual Distress Scale – Revised

**Fig. 3:**
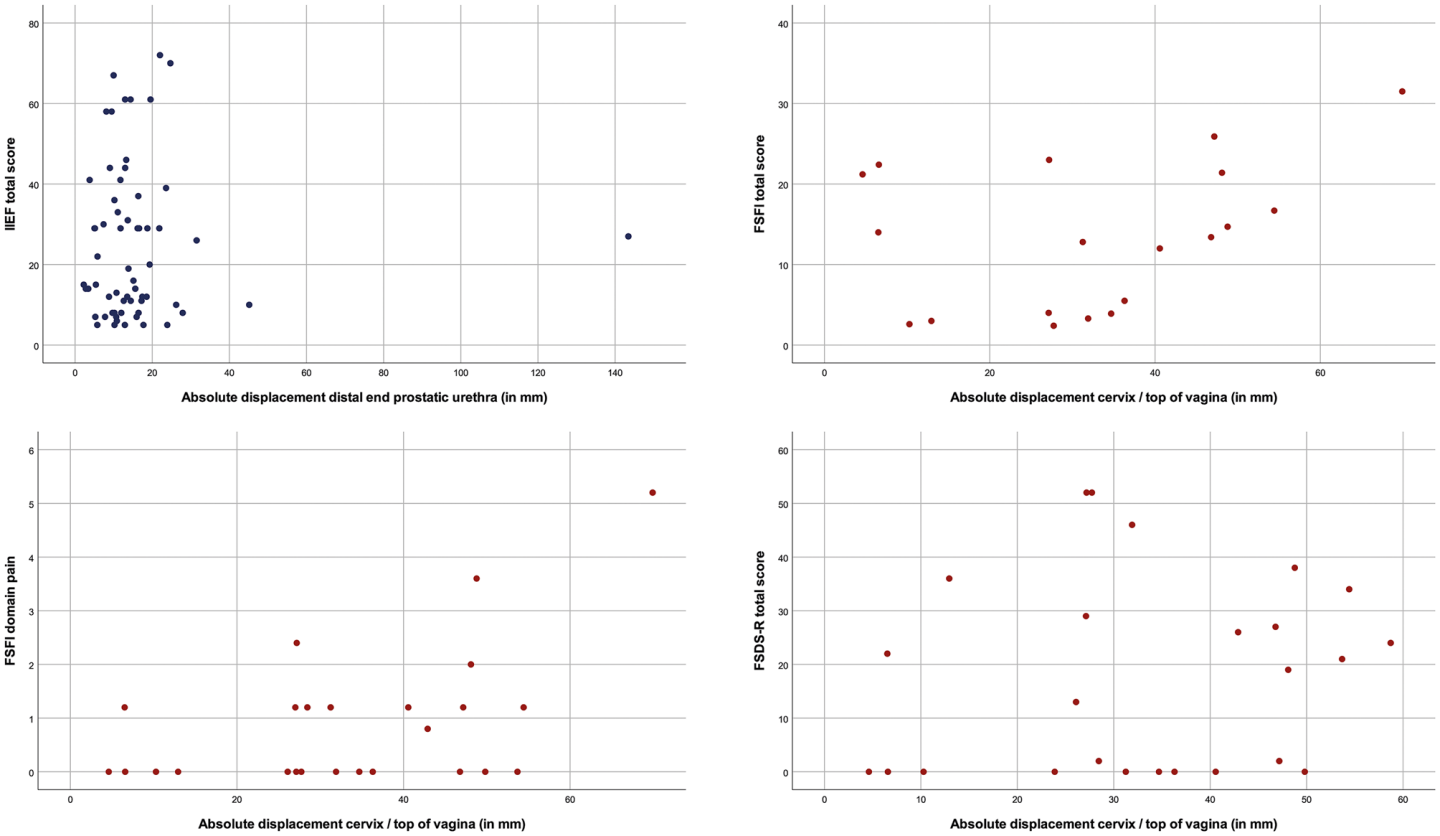
Scatterplots: all patients. Correlation of the absolute displacement in millimeters of the distal end of the prostatic urethra with IIEF total score. Correlation of the absolute displacement in millimeters of the cervix/top of vagina with FSFI total score, FSFI domain pain, and FSDS-R total score

**Fig. 4:**
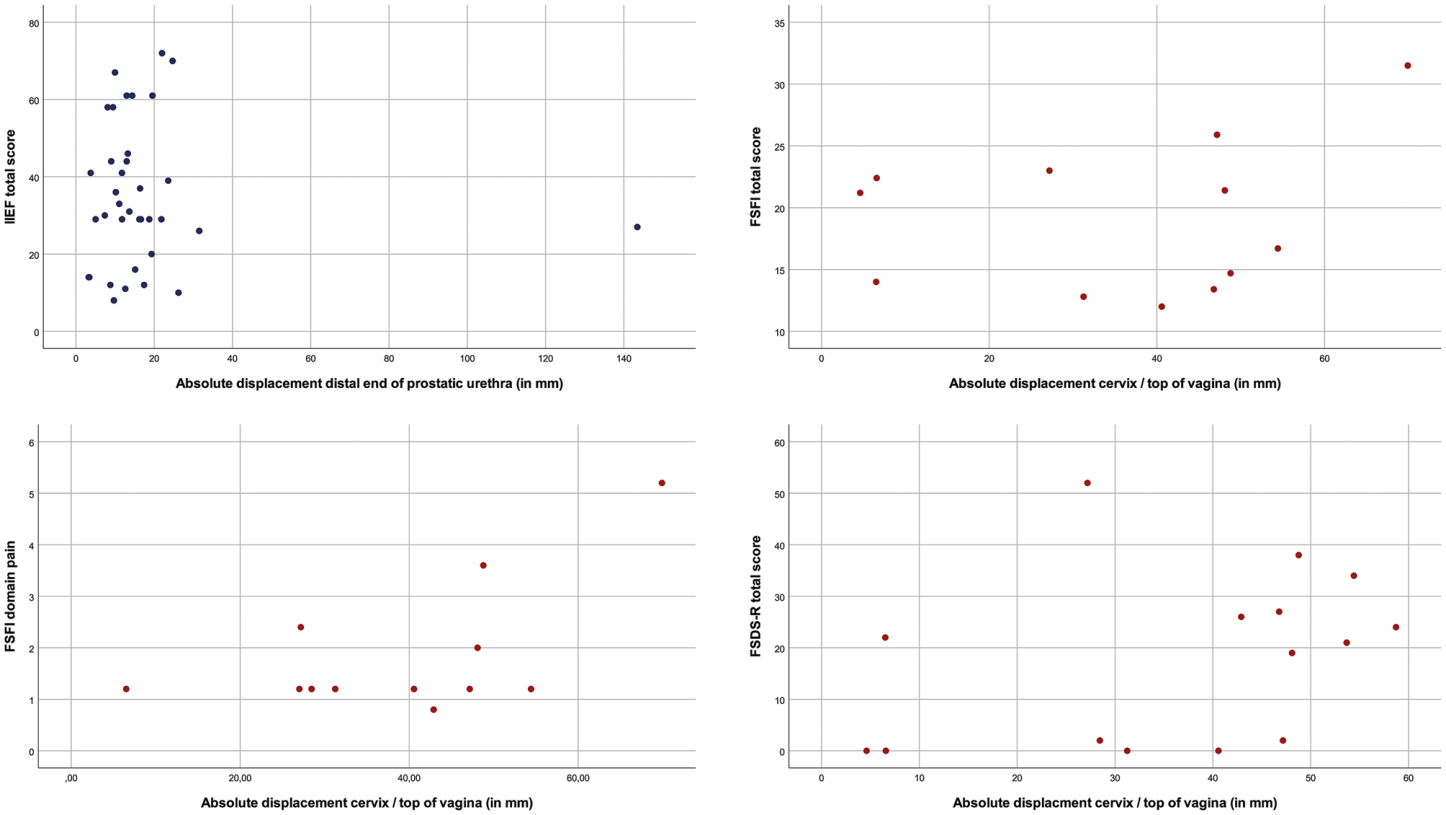
Scatterplots: sexually active patients. Correlation of the absolute displacement in millimeters of the distal end of the prostatic urethra with IIEF total score. Correlation of the absolute displacement in millimeters of the cervix/top of vagina with FSFI total score, FSFI domain pain, and FSDS-R total score

**Fig. 5:**
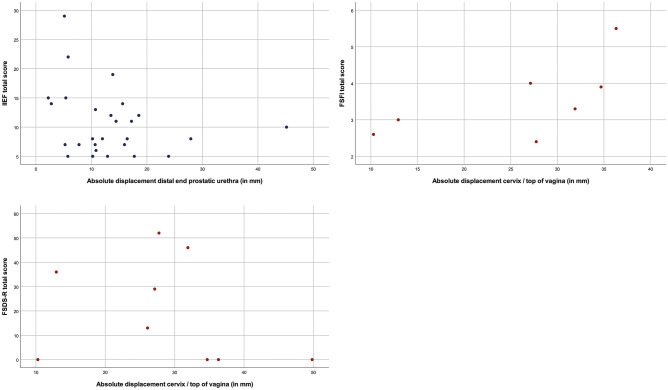
Scatterplots: sexually inactive patients. Correlation of the absolute displacement in millimeters of the distal end of the prostatic urethra with IIEF total score. Correlation of the absolute displacement in millimeters of the cervix/top of vagina with FSFI total score and FSDS-R total score

In women with minimal and substantial displacement of the internal urethral orifice, median UDI-6 scores were 25 (IQR 10–46) and 21 (IQR 16–36), respectively (*P* = 0.827). Corresponding scores in men were 10 (IQR 0–22) and 17 (IQR 5–21) (*P* = 0.330). Similar outcomes were found regarding the landmark posterior bladder wall (Table 3).

**Table 3:** Mann–Whitney *U* test comparing subgroups divided by median split

**Gender**	**Landmark**	**Questionnaire**		**Minimal displacement**		**Substantial displacement**		***P*-value**
			**Domain**	***n***	**Median score (IQR)**	***n***	**Median score (IQR)**	
♂	Internal urethral orifice	UDI-6		38	10 (0–22)	44	17 (5–21)	0.330
♂	Internal urethral orifice	UDI-6	Obstructive	39	13 (0–25)	44	13 (0–25)	0.935
♂	Internal urethral orifice	IIQ-7		39	0 (0–10)	45	0 (0–14)	0.524
♀	Internal urethral orifice	UDI-6		17	25 (10–46)	14	21 (16–36)	0.827
♀	Internal urethral orifice	UDI-6	Obstructive	17	25 (0–38)	14	19 (9–38)	0.776
♀	Internal urethral orifice	IIQ-7		16	7 (0–32)	14	14 (0–35)	0.728
♂	Posterior bladder wall	UDI-6		39	13 (4–25)	43	17 (4–21)	0.955
♂	Posterior bladder wall	UDI-6	Obstructive	40	13 (0–25)	43	13 (0–13)	0.626
♂	Posterior bladder wall	IIQ-7		42	0 (0–29)	42	0 (0–5)	0.545
♀	Posterior bladder wall	UDI-6		17	25 (10–44)	16	21 (14–32)	0.504
♀	Posterior bladder wall	UDI-6	Obstructive	17	25 (6–38)	16	13 (0–38)	0.416
♀	Posterior bladder wall	IIQ-7		17	10 (0–31)	16	14 (0–33)	0.910
♂	Distal end of prostatic urethra	IIEF		33	15 (8–39)	30	20 (11–33)	0.725
♀	Cervix/top of vagina	FSFI		10	8 (3–22)	9	15 (9–24)	0.165
♀	Cervix/top of vagina	FSFI	Pain	5	1 (1–2)	7	1 (1–4)	0.651
♀	Cervix/top of vagina	FSDS-R		13	13 (0–41)	12	20 (0–27)	0.759

*UDI-6* Urogenital Distress Inventory short form, *IIQ-7* Incontinence Impact Questionnaire short form, *IIEF* International Index of Erectile Function, *FSFI* Female Sexual Function Index (converted), *FSDS-R* Female Sexual Distress Scale – Revised

#### Sexual function

Before APR, mean sexual interest scores were 71.6 ± 28.8 in evaluable male and 91.7 ± 15.4 in female patients. Mean impotence score was 29.2 ± 33.1, and mean dyspareunia score was 0.0 ± 0.0 (Supplementary Table S7).

At time the postoperative questionnaire was completed, 52% (45/87) of male and 59% (19/32) of female patients were sexually active (*P* = 0.458), and 31% (26/85) and 46% (13/28) engaged in intercourse (*P* = 0.126), respectively. There was no difference in the proportion of women having intercourse between those with minimal (42%, 5/12) and substantial displacement (58%, 7/12; *P* = 0.320).

The absolute displacement of the distal end of the prostatic urethra was not correlated with IIEF (*ρ* = 0.023, *P* = 0.855) overall, neither in sexually active patients (*ρ* = 0.015, *P* = 0.929). A correlation in contrary direction was found between absolute displacement of the cervix and FSFI (*ρ* = 0.321, *P* = 0.180), which became stronger in sexually inactive patients (*ρ* = 0.607, *P* = 0.148). Also regarding the domain pain, contrary outcomes were found (*ρ* = 0.390, *P* = 0.210). The absolute displacement of the cervix was correlated with FSDS-R (*ρ* = 0.433, *P* = 0.107) in sexually active patients. Table 2 presents the Spearman’s Rho analyses, and scatterplots are illustrated in Figs. 3a-d (total), 4a–d (sexually active), and 5a–c (sexually inactive).

In women, median FSDS-R scores were higher, implying more sexual dysfunction, for substantial displacement of the cervix 20 (IQR 0–27) and 13 (IQR 0–41; *P* = 0.759). Contrary outcomes were found when using FSFI to examine the degree of sexual dysfunction (8 (IQR 3–22) vs. 15 (IQR 9–24); *P* = 0.165). Also in men, median IIEF scores were higher, indicating less sexual dysfunction, for substantial displacement of the distal end of the prostatic urethra (20 (IQR 11–33) vs. 15 (IQR 8–39); *P* = 0.725) (Table 3).

### Rotation

The Spearman’s Rho of the correlation between the rotation of the different anatomical landmarks and urogenital questionnaires is presented in Supplementary Table S8 and illustrated in Scatterplots in Supplementary Figures S2a–f, S3a–d (total), S4a–d (sexually active), and S5a–c (sexually inactive).

## Discussion

Wide variety in caudo-dorsal displacement of the bladder and internal genital organs with upper limits of almost 10 cm were observed after APR, and based on clinical observations in routine daily practice, patients might have related functional complaints (e.g., overflow incontinence, no vaginal penetration possible). However, we were not able to correlate the degree of urethral and bladder displacement with scores of validated urinary questionnaires in male and female patients. Findings supporting our hypothesis were correlations between absolute displacement of the cervix and FSDS-R score (*ρ* = 0.433) in sexually active patients. Furthermore, median FSDS-R score in women with substantial displacement of the cervix was seven points higher if compared to minimal displacement, which was higher than the MCID of six.

The IIQ-7 questionnaire focuses on the consequences of urinary leakage for daily life, whereas the UDI-6 questionnaire focuses more specifically on different types of urinary complaints. We hypothesized that caudo-dorsal displacement of the bladder after removal of the rectum with a concomitant risk of autonomic nerve injury could result in bladder emptying difficulties, and this might be reflected by the domain obstructive. However, when analyzing this domain separately, no correlation with displacement of the bladder was found. This might be explained by only one (question 5) of the two questions of this domain being relevant.

We used the IIEF questionnaire for men, although this questionnaire was designed to examine the effectiveness of sildenafil on erectile function and focused on sexual intercourse [11]. Therefore, this questionnaire reflects the answer ‘’no sexual activity’’ as poor sexual function. We analyzed patients with and without sexual activity separately, since the reason for ‘’no sexual activity’’ is unclear, e.g., not having a partner or already not being sexually active before APR. Also, patients may have lost interest in sex and are therefore not sexually active, which does not directly imply that they have poor sexual function. Furthermore, the magnitude of displacement might reflect the extent of the surgical resection. A larger resection might cause more autonomic nerve damage, which is related to erectile function and could have influenced our results.

The FSDS-R questionnaire measures the extent to which women experience personal distress about their sexual problems in daily life, whereas the FSFI questionnaire focused on specific aspects of sexual dysfunction. Comparable to the IIEF in men, the FSFI reflects ‘’no sexual activity’’ as poor sexual function, and therefore we analyzed these patients separately. Displacement of the cervix may represent angulation of the vagina, after which intercourse might be painful or even no longer possible. Therefore, we examined the domain pain of FSFI separately. However, a correlation in contradictory direction was found, probably due to very low number of patients. Regarding FSDS-R, a higher degree of displacement resulted in more dysfunction in sexually active patients. In addition, an MCID was reached when the displacement was divided into minimal and substantial displacement. Therefore, the FSDS-R questionnaire might be more appropriate for this patient group, since the FSFI questionnaire focused on being sexually active and intercourse.

Several studies investigated the urogenital function after rectal surgery [1, 13–15]. So far, these studies did not examine the correlation between urogenital function and the magnitude of displacement of urogenital organs. One study investigated the urogenital function after APR for rectal cancer; however, this study did not use validated questionnaires [1]. Therefore, we cannot compare our scores. Another study used the FSFI questionnaire in patients who underwent APR for squamous cell carcinoma of the anus. However, the difference in patient groups makes it difficult to compare our results [16]. Regarding the number of sexually active patients, we found a higher rate compared to a former study presenting quality of life in patients following APR for rectal cancer (59% vs. 17%) [17]. This difference might be explained by the high drop-out rate in our study, but also the return of questionnaires might be biased by sexual activity. Patients who are sexually active are probably more likely to respond to the questionnaires. Regarding confounding factors, one study found that perioperative blood loss was significantly associated with urinary dysfunction after APR [15]. Other associated factors were preoperative difficulty in bladder emptying and autonomic never damage. However, since we were not able to correlate the degree of urethral displacement with scores of validated urinary questionnaires in both genders, there was no utility for multivariate testing.

An important limitation of this study is the retrospective design. Therefore we could not assess the difference between urogenital function pre- and postoperatively. The time interval between surgery and completion of questionnaires was median more than 4 years; thus, some degenerative mechanism due to aging might have influenced the outcomes. In addition, the questionnaires were not designed for this patient group since the reason for sexual inactivity was not addressed. Sexual function is a confidential topic which is represented in the low response rate causing selection bias. The relatively small number of patients in analyses of specific domains of the questionnaires makes it difficult to draw firm conclusions. Furthermore, the sexual condition of the partner was not taken into account in the questionnaires. Regarding the correlation with the displacement, the radiological scans were performed in supine position without active pressure, while patients mainly experience urogenital dysfunction in standing position and with straining. Literature reveals that upright MRI scanning and straining both show a larger extent of prolapse [18, 19].

Regarding the clinical implications, this study creates awareness to inform patients decently in advance, and that attention should be paid to possible changes in urogenital function. Future studies might focus on the development of more specific questionnaires for this patient cohort. In addition, preoperative radiotherapy and pre-existing comorbidities are important potential confounders and should be incorporated in future research that analyzes the impact of urogenital organ displacement on urinary and sexual function.

## Conclusion

This study examined the use of validated questionnaires to objectify the functional impact of displacement of urogenital organs after APR for rectal cancer. In female patients, the degree of cervix displacement and the scores of relevant sexual questionnaires seemed to be correlated. The data did not indicate any functional impact of bladder displacement, potentially explained by the urinary questionnaires that we used. This first explorative analysis can generate hypotheses to further explore this underexposed topic in larger prospective studies.

## Supplementary Information

Below is the link to the electronic supplementary material.

Supplementary file1 (TIF 13674 KB)

Supplementary file2 (TIF 27588 KB)

Supplementary file3 (TIF 53376 KB)

Supplementary file4 (TIF 52952 KB)

Supplementary file5 (TIF 53937 KB)

Supplementary file6 (DOCX 13 KB)

Supplementary file7 (DOCX 13 KB)

Supplementary file8 (DOCX 16 KB)

Supplementary file9 (DOCX 17 KB)

Supplementary file10 (DOCX 15 KB)

Supplementary file11 (DOCX 20 KB)

Supplementary file12 (DOCX 13 KB)

Supplementary file13 (DOCX 16 KB)

## Data Availability

The data that support the findings of this study are available from the corresponding author.
