# Supplementary figures and images for: Exploring the impact of urogenital organ displacement after abdominoperineal resection on urinary and sexual function
Source: Int J Colorectal Dis. 2022 Aug 31;37(10):2125–36. doi: 10.1007/s00384-022-04234-3 (PMC9562368; doi:10.1007/s00384-022-04234-3)

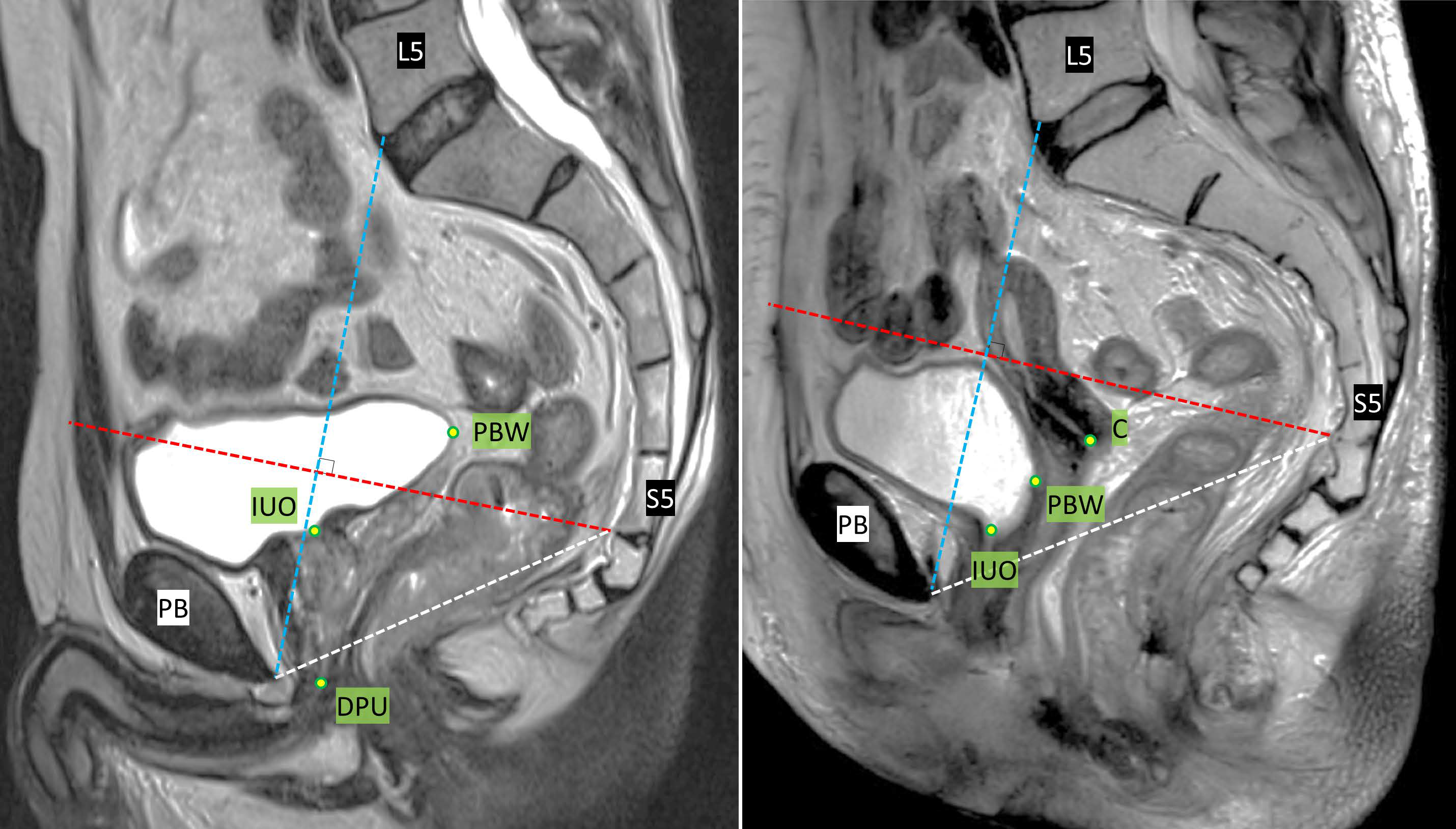

Supplement: Supplementary file 1 — Supplementary file1 (TIF 13674 KB) [file 384_2022_4234_MOESM1_ESM.tif]

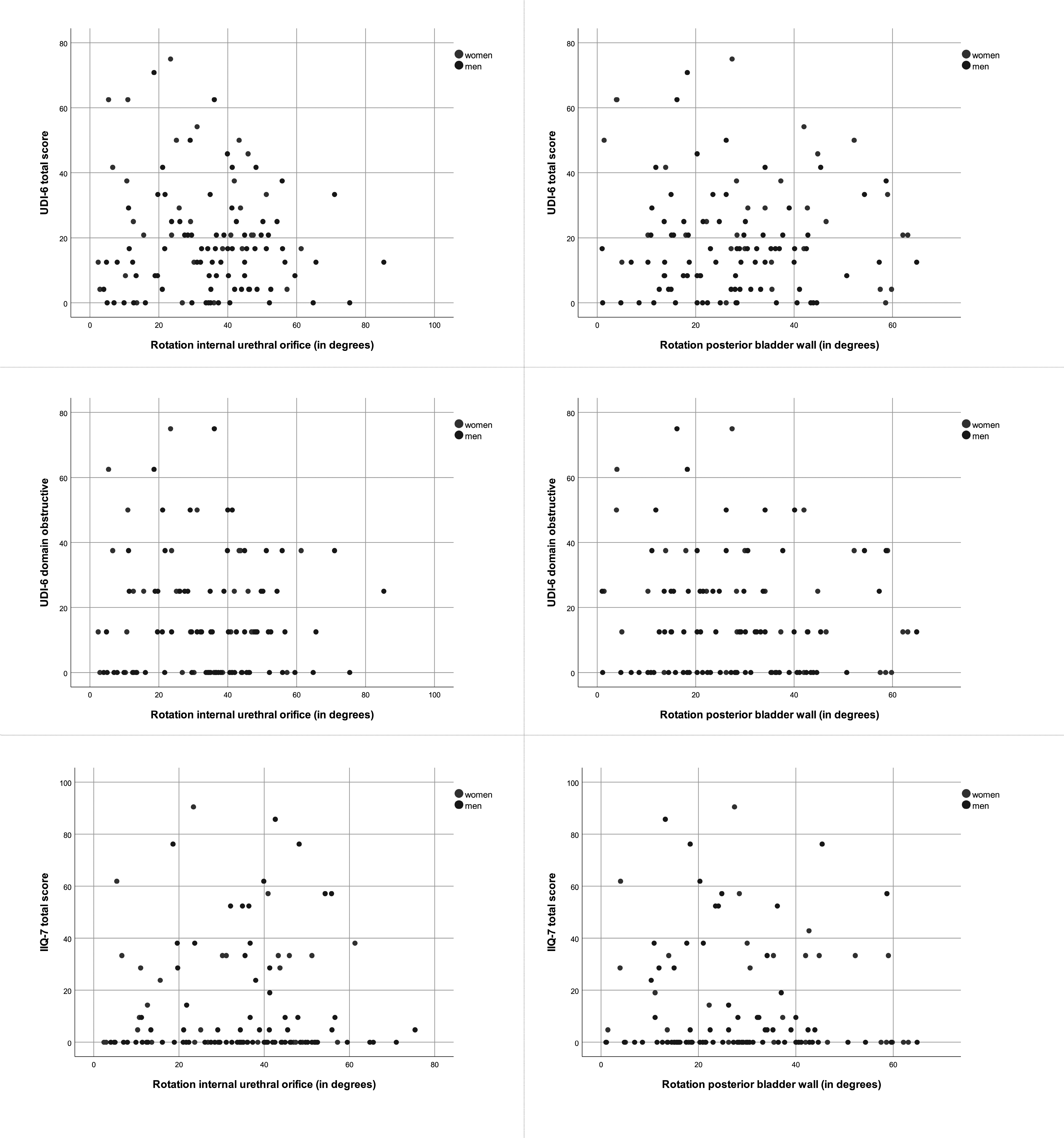

Supplement: Supplementary file 2 — Supplementary file2 (TIF 27588 KB) [file 384_2022_4234_MOESM2_ESM.tif]

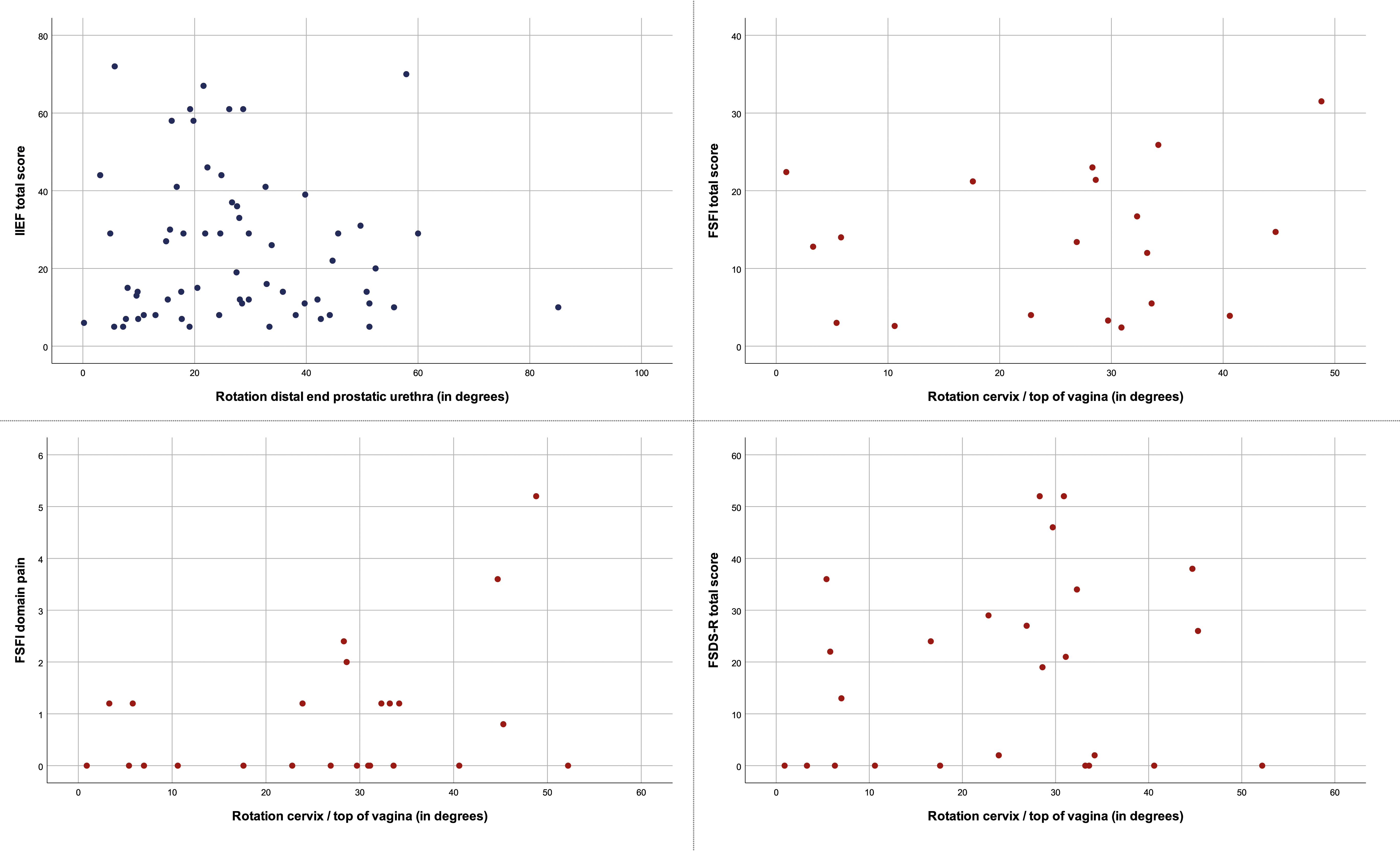

Supplement: Supplementary file 3 — Supplementary file3 (TIF 53376 KB) [file 384_2022_4234_MOESM3_ESM.tif]

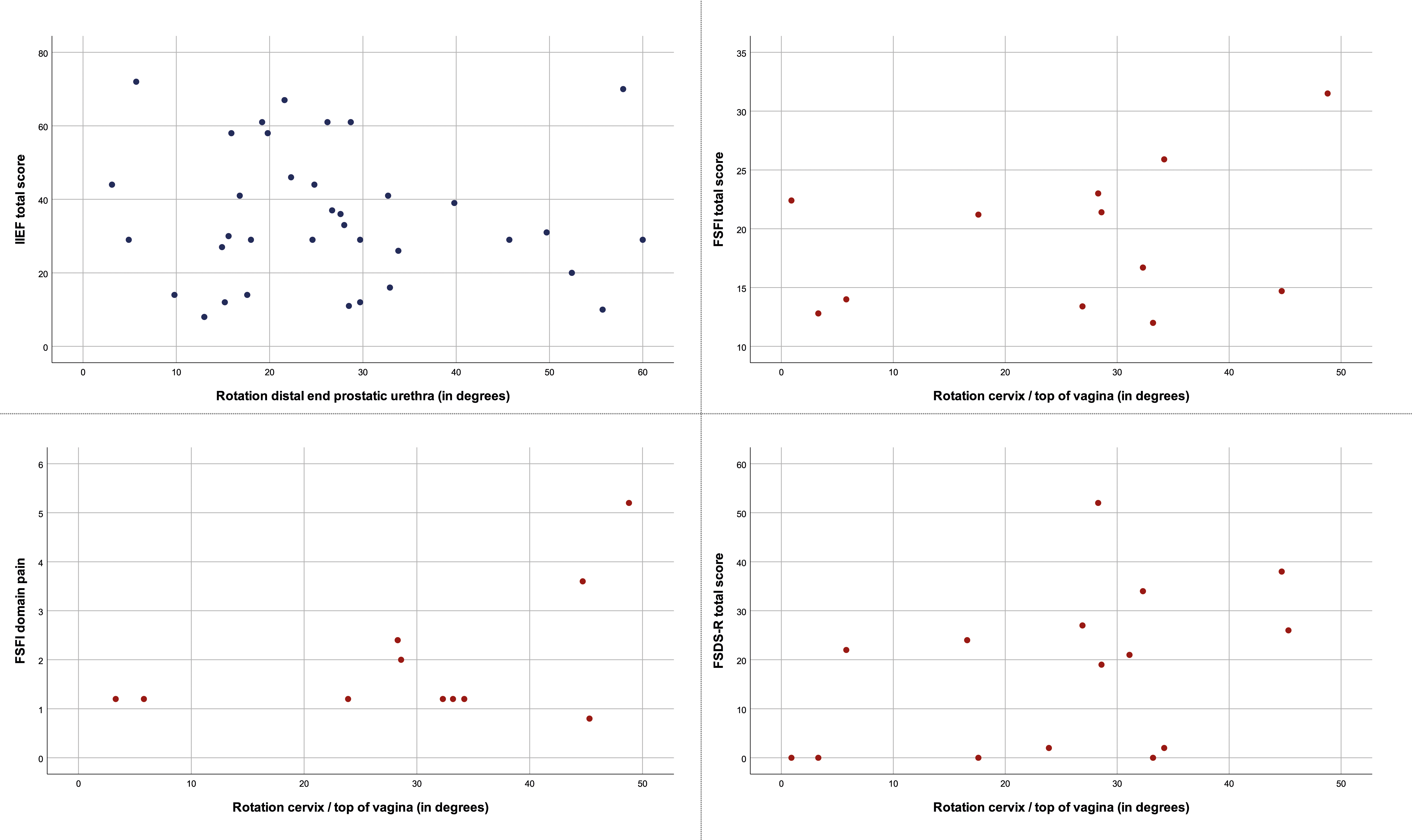

Supplement: Supplementary file 4 — Supplementary file4 (TIF 52952 KB) [file 384_2022_4234_MOESM4_ESM.tif]

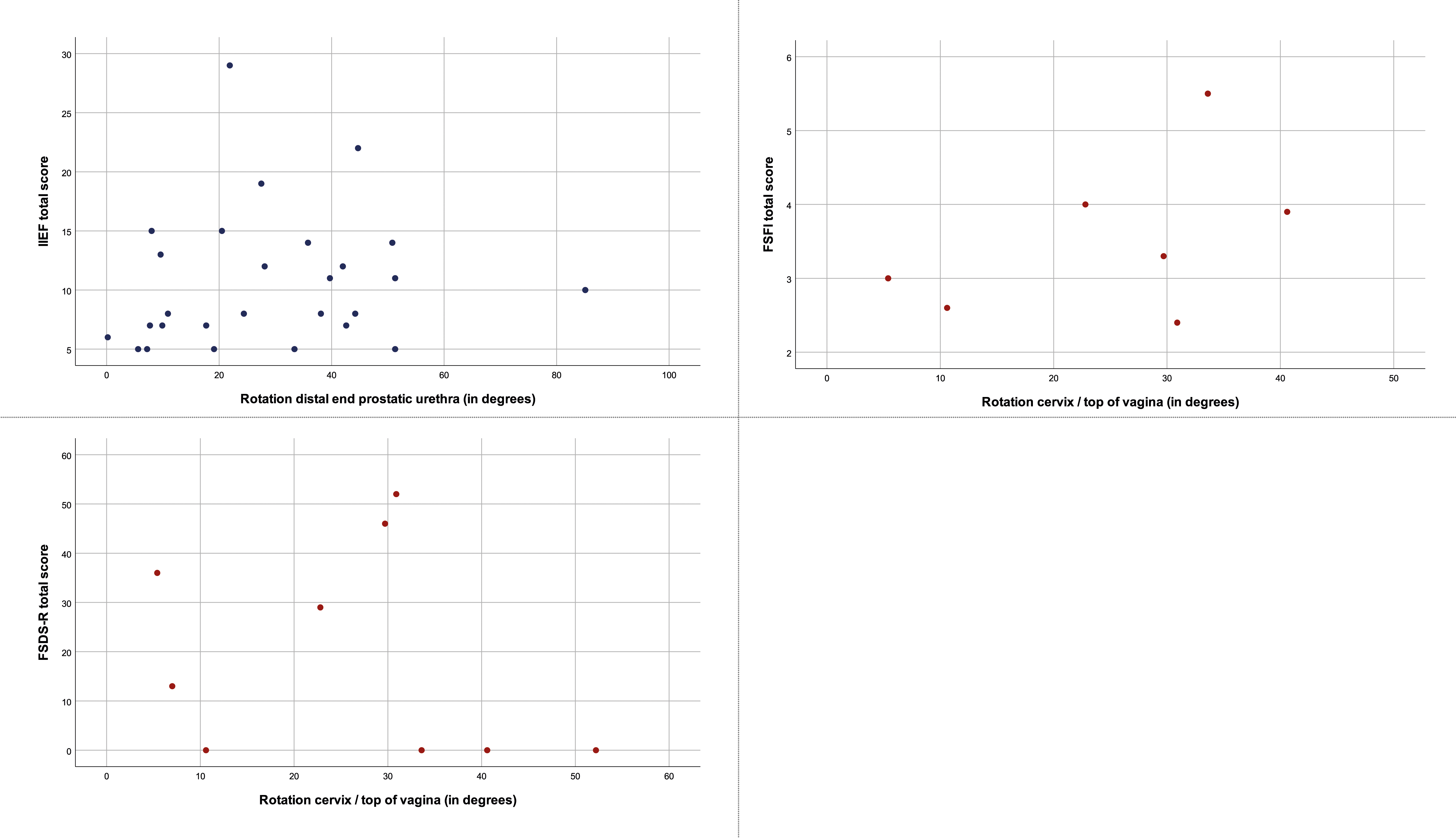

Supplement: Supplementary file 5 — Supplementary file5 (TIF 53937 KB) [file 384_2022_4234_MOESM5_ESM.tif]
