# Supplementary material for: Exploring the impact of urogenital organ displacement after abdominoperineal resection on urinary and sexual function
Source: Int J Colorectal Dis. 2022 Aug 31;37(10):2125–36. doi: 10.1007/s00384-022-04234-3 (PMC9562368; doi:10.1007/s00384-022-04234-3)
Supplement: Supplementary file 6 — Supplementary file6 (DOCX 13 KB) [file 384_2022_4234_MOESM6_ESM.docx]

**Supplementary Table 1** Urogenital Distress Inventory (UDI-6)

Do you experience? If so how much are you bothered by:

|  |  | Not at all | A little bit | Moderately | Greatly |
| --- | --- | --- | --- | --- | --- |
|  |  |  |  |  |  |
| 1. | Frequent urination? | 0 | 1 | 2 | 3 |
|  |  |  |  |  |  |
| 2. | Urine leakage related to the feeling of | 0 | 1 | 2 | 3 |
| urgency? | |  |  |  |  |
| 3. | Urine leakage related to physical activity, | 0 | 1 | 2 | 3 |
| coughing or sneezing (that it drops)? | |  |  |  |  |
|  |  |  |  |  |  |
| 4. | Small amounts of urine leakage (that it | 0 | 1 | 2 | 3 |
| drops)? | |  |  |  |  |
| 5. | Difficulty emptying your bladder? | 0 | 1 | 2 | 3 |
|  |  |  |  |  |  |
| 6. | Pain or discomfort in the lower abdominal | 0 | 1 | 2 | 3 |
| or genital area? | |  |  |  |  |
|  |  |  |  |  |  |
