# Supplementary material for: Exploring the impact of urogenital organ displacement after abdominoperineal resection on urinary and sexual function
Source: Int J Colorectal Dis. 2022 Aug 31;37(10):2125–36. doi: 10.1007/s00384-022-04234-3 (PMC9562368; doi:10.1007/s00384-022-04234-3)
Supplement: Supplementary file 7 — Supplementary file7 (DOCX 13 KB) [file 384_2022_4234_MOESM7_ESM.docx]

**Supplementary Table 2** Incontinence Impact Questionnaire – Short Form IIQ-7

Some people find that accidental urine loss may affect their activities, relationships, and feelings. The questions below refer to areas in your life that may have been influenced or changed by your problem. For each question, circle the response that best describes how much your activities, relationships, and feelings are being affected by urine leakage.

Has urine leakage affected your:

|  |  | Not at all | Slightly | Moderately | Greatly |
| --- | --- | --- | --- | --- | --- |
| 1. | Ability to do household chores (cooking, | 0 | 1 | 2 | 3 |
| housecleaning, laundry)? | |  |  |  |  |
| 2. | Physical recreation such as walking, | 0 | 1 | 2 | 3 |
| swimming or other exercise? | |  |  |  |  |
| 3. | Entertainment activities (movies, concerts, | 0 | 1 | 2 | 3 |
| etc.)? | |  |  |  |  |
| 4. | Ability to travel by car or bus more than 30 | 0 | 1 | 2 | 3 |
| minutes from home? | |  |  |  |  |
| 5. | Participation in social activities outside your | 0 | 1 | 2 | 3 |
| home? | |  |  |  |  |
| 6. | Emotional health (nervousness, depression, | 0 | 1 | 2 | 3 |
| etc.)? | |  |  |  |  |
| 7. | Feeling frustrated? | 0 | 1 | 2 | 3 |
