# Supplementary material for: Exploring the impact of urogenital organ displacement after abdominoperineal resection on urinary and sexual function
Source: Int J Colorectal Dis. 2022 Aug 31;37(10):2125–36. doi: 10.1007/s00384-022-04234-3 (PMC9562368; doi:10.1007/s00384-022-04234-3)
Supplement: Supplementary file 8 — Supplementary file8 (DOCX 16 KB) [file 384_2022_4234_MOESM8_ESM.docx]

**Supplementary Table 3** International Index of Erectile Function (IIEF)

These questions ask about the effects that your erection problems have had on your sex life over the last four weeks. Please try to answer the questions as honestly and as clearly as you are able. In answering the questions, the following definitions apply:

- **Sexual activity** includes intercourse, caressing, foreplay and masturbation
- **Sexual intercourse** is defined as sexual penetration of your partner
- **Sexual stimulation** is the ejection of semen from the penis (or the feeling of this)
- **Orgasm** is the fulfilment or climax following sexual stimulation or intercourse

Over the past 4 weeks: *please check one box*

1. How often were you able to get an erection during sexual activity?

- No sexual activity
- Almost never or never
- A few times (less than half the time)
- Sometimes (about half the time)
- Most times (more than half the time)
- Almost always or always

1. When you had erections with sexual stimulation, how often were your erections hard enough for penetration?

- No sexual activity
- Almost never or never
- A few times (less than half the time)
- Sometimes (about half the time)
- Most times (more than half the time)
- Almost always or always

1. When you attempted intercourse, how often were you able to penetrate (enter) your partner?

- Did not attempt intercourse
- Almost never or never
- A few times (less than half the time)
- Sometimes (about half the time)
- Most times (more than half the time)
- Almost always or always

1. During sexual intercourse, how often were you able to maintain your erection after you had penetrated (entered) your partner?

- Did not attempt intercourse
- Almost never or never
- A few times (less than half the time)
- Sometimes (about half the time)
- Most times (more than half the time)
- Almost always or always

1. During sexual intercourse, how difficult was it to maintain your erection to completion of intercourse?

- Did not attempt intercourse
- Extremely difficult
- Very difficult
- Difficult
- Slightly difficult
- Not difficult

1. How many times have you attempted sexual intercourse?

- No attempts
- One to two attempts
- Three to four attempts
- Five to six attempts
- Seven to ten attempts
- Eleven or more attempts

1. When you attempted sexual intercourse, how often was it satisfactory for you?

- Did not attempt intercourse
- Almost never or never
- A few times (less than half the time)
- Sometimes (about half the time)
- Most times (more than half the time)
- Almost always or always

1. How much have you enjoyed sexual intercourse?

- No intercourse
- No enjoyment at all
- Not very enjoyable
- Fairly enjoyable
- Highly enjoyable
- Very highly enjoyable

1. When you had sexual stimulation or intercourse, how often did you ejaculate?

- Did not attempt intercourse
- Almost never or never
- A few times (less than half the time)
- Sometimes (about half the time)
- Most times (more than half the time)
- Almost always or always

1. When you had sexual stimulation or intercourse, how often did you have the feeling of orgasm or climax?

- Almost never or never
- A few times (less than half the time)
- Sometimes (about half the time)
- Most times (more than half the time)
- Almost always or always

1. How often have you felt sexual desire?

- Almost never or never
- A few times (less than half the time)
- Sometimes (about half the time)
- Most times (more than half the time)
- Almost always or always

1. How would you rate your level of sexual desire?

- Very low or none at all
- Low
- Moderate
- High
- Very high

1. How satisfied have you been with your overall sex life?

- Very dissatisfied
- Moderately dissatisfied
- Equally satisfied and dissatisfied
- Moderately satisfied
- Very satisfied

1. How satisfied have you been with your sexual relationship with your partner?

- Very dissatisfied
- Moderately dissatisfied
- Equally satisfied and dissatisfied
- Moderately satisfied
- Very satisfied

1. How do you rate your confidence that you could get and keep an erection?

- Very low
- Low
- Moderate
- High
- Very high
