# Supplementary material for: Exploring the impact of urogenital organ displacement after abdominoperineal resection on urinary and sexual function
Source: Int J Colorectal Dis. 2022 Aug 31;37(10):2125–36. doi: 10.1007/s00384-022-04234-3 (PMC9562368; doi:10.1007/s00384-022-04234-3)
Supplement: Supplementary file 9 — Supplementary file9 (DOCX 17 KB) [file 384_2022_4234_MOESM9_ESM.docx]

**Supplementary Table 4** Female Sexual Function Index (FSFI)

INSTRUCTIONS: These questions ask about your sexual feelings and responses during the past 4 weeks. Please answer the following questions as honestly and clearly as possible. Your responses will be kept completely confidential. In answering these questions the following definitions apply:

Sexual activity can include caressing, foreplay, masturbation and vaginal intercourse.

Sexual intercourse is defined as penile penetration (entry) of the vagina.

Sexual stimulation includes situations like foreplay with a partner, self-stimulation (masturbation), or sexual fantasy.

CHECK ONLY ONE BOX PER QUESTION.

Sexual desire or interest is a feeling that includes wanting to have a sexual experience, feeling receptive to a partner's sexual initiation, and thinking or fantasizing about having sex.

1. Over the past 4 weeks, how **often** did you feel sexual desire or interest?

- Almost always or always
- Most times (more than half the time)
- Sometimes (about half the time)
- A few times (less than half the time)
- Almost never or never

1. Over the past 4 weeks, how would you rate your **level** (degree) of sexual desire or interest?

- Very high
- High
- Moderate
- Low
- Very low or none at all

Sexual arousal is a feeling that includes both physical and mental aspect of sexual excitement. It may include feelings of warmth or tingling in the genitals, lubrication (wetness), or muscle contractions.

1. Over the past 4 weeks, how **often** did you feel sexually aroused (‘’turned on’’) during sexual activity or intercourse?

- No sexual activity
- Almost always or always
- Most times (more than half the time)
- Sometimes (about half the time)
- A few times (less than half the time)

1. Over the past 4 weeks, how would you rate your **level** of sexual arousal (‘’turn on’’) during sexual activity or intercourse?

- No sexual activity
- Very high
- High
- Moderate
- Low
- Very low or none at all

1. Over the past 4 weeks, how **confident** were you about becoming sexually aroused during sexual activity or intercourse?

- No sexual activity
- Very high confidence
- High confidence
- Moderate confidence
- Low confidence
- Very low confidence

1. Over the past 4 weeks, how often have you been satisfied with your arousal (excitement) during sexual activity or intercourse?

- No sexual activity
- Almost always or always
- Most times (more than half the time)
- Sometimes (about half the time)
- A few times (less than half the time)
- Almost never or never

1. Over the past 4 weeks, how **often** did you become lubricated (‘’wet’’) during sexual activity or intercourse?

- No sexual activity
- Almost always or always
- Most times (more than half the time)
- Sometimes (about half the time)
- A few times (less than half the time)

1. Over the past 4 weeks, how **difficult** was it to become lubricated (‘’wet’’) during sexual activity or intercourse?

- No sexual activity
- Extremely difficult or impossible
- Very difficult
- Difficult
- Slightly difficult
- Not difficult

1. Over the past 4 weeks, how often did you **maintain** your lubrication (‘’wetness’’) until completion of sexual activity or intercourse?

- No sexual activity
- Almost always or always
- Most times (more than half the time)
- Sometimes (about half the time)
- A few times (less than half the time)
- Almost never or never

1. Over the past 4 weeks, how **difficult** was it to maintain your lubrication (‘’wetness’’) until completion of sexual activity or intercourse?

- No sexual activity
- Extremely difficult or impossible
- Very difficult
- Difficult
- Slightly difficult
- Not difficult

1. Over the past 4 weeks, when you had sexual stimulation or intercourse, how **often** did you reach orgasm (climax)?

- No sexual activity
- Almost always or always
- Most times (more than half the time)
- Sometimes (about half the time)
- A few times (less than half the time)
- Almost never or never

1. Over the past 4 weeks, when you had sexual stimulation or intercourse, how **difficult** was it for you to reach orgasm (climax)?

- No sexual activity
- Extremely difficult or impossible
- Very difficult
- Difficult
- Slightly difficult
- Not difficult

1. Over the past 4 weeks, how **satisfied** were you with your ability to reach orgasm (climax) during sexual activity or intercourse?

- No sexual activity
- Very satisfied
- Moderately satisfied
- About equally satisfied an dissatisfied
- Moderately dissatisfied
- Very dissatisfied

1. Over the past 4 weeks, how **satisfied** have you been with the amount of emotional closeness during sexual activity between you and your partner?

- No sexual activity
- Very satisfied
- Moderately satisfied
- About equally satisfied and dissatisfied
- Moderately dissatisfied
- Very dissatisfied

1. Over the past 4 weeks, how **satisfied** have you been with your sexual relationship with your partner?

- Very satisfied
- Moderately satisfied
- About equally satisfied and dissatisfied
- Moderately dissatisfied
- Very dissatisfied

1. Over the past 4 weeks, how **satisfied** have you been with your overall sexual life?

- Very satisfied
- Moderately satisfied
- About equally satisfied and dissatisfied
- Moderately dissatisfied
- Very dissatisfied

1. Over the past 4 weeks, how **often** did you experience discomfort or pain during vaginal penetration?

- Did not attempt intercourse
- Almost always or always
- Most times (more than half the time)
- Sometimes (about half the time)
- A few times (less than half the time)
- Almost never or never

1. Over the past 4 weeks, how **often** did you experience discomfort or pain following vaginal penetration?

- Did not attempt intercourse
- Almost always or always
- Most times (more than half the time)
- Sometimes (about half the time)
- A few times (less than half the time)
- Almost never or never

1. Over the past 4 weeks, how would you rate your **level** (degree) of discomfort or pain during or following vaginal penetration?

- Did not attempt intercourse
- Very high
- High
- Moderate
- Low
- Very low or none at all
