# Supplementary material for: Exploring the impact of urogenital organ displacement after abdominoperineal resection on urinary and sexual function
Source: Int J Colorectal Dis. 2022 Aug 31;37(10):2125–36. doi: 10.1007/s00384-022-04234-3 (PMC9562368; doi:10.1007/s00384-022-04234-3)
Supplement: Supplementary file 10 — Supplementary file10 (DOCX 15 KB) [file 384_2022_4234_MOESM10_ESM.docx]

**Supplementary Table 5** Female Sexual Distress Scale

Below is a list of feelings and problems that women sometimes have concerning their sexuality. Please read each item carefully, and circle the number that best describes HOW OFTEN THAT PROBLEM HAS BOTHERED YOU OR CAUSED YOU DISTRESS DURING THE PAST 30 DAYS INCLUDING TODAY.

Circle only one number for each item, and take care not to skip any items. If you change your mind, erase your first circle carefully. Read the example before beginning, and if you have any questions please ask about them.

Example: How often did you feel: **Personal responsibility for your sexual problems.**

|  | Never | | |  | Rarely |  | Occasionally | |  | Frequently | | |  |  |  | Always | | |
| --- | --- | --- | --- | --- | --- | --- | --- | --- | --- | --- | --- | --- | --- | --- | --- | --- | --- | --- |
|  |  | 0 |  |  | 1 |  | 2 |  |  |  | 3 | |  |  |  | 4 |  |  |
|  | **How often did you feel:** | | | | | |  |  |  |  |  |  |  |  |  |  |  |  |
|  |  |  | | | | | | |  | |  |  |  |  |  |  |  |  |
|  | 1. | Distressed about your sex life | | | | | | | 0 | |  | 1 |  | 2 | 3 |  | 4 |  |
|  | 2. | Unhappy about your sexual relationship | | | | | | | 0 | |  | 1 |  | 2 | 3 |  | 4 |  |
|  | 3. | Guilty about sexual difficulties | | | | | | | 0 | |  | 1 |  | 2 | 3 |  | 4 |  |
|  | 4. | Frustrated by your sexual problems | | | | | | | 0 | |  | 1 |  | 2 | 3 |  | 4 |  |
|  | 5. | Stressed about sex | | | | |  |  | 0 | |  | 1 |  | 2 | 3 |  | 4 |  |
|  | 6. | Inferior because of sexual problems | | | | | | | 0 | |  | 1 |  | 2 | 3 |  | 4 |  |
|  | 7. | Worried about sex | | | | |  |  | 0 | |  | 1 |  | 2 | 3 |  | 4 |  |
|  | 8. | Sexually inadequate | | | | |  |  | 0 | |  | 1 |  | 2 | 3 |  | 4 |  |
|  | 9. | Regrets about your sexuality | | | | | | | 0 | |  | 1 |  | 2 | 3 |  | 4 |  |
|  | 10. | | Embarrassed about sexual problems | | | | | | 0 | |  | 1 |  | 2 | 3 |  | 4 |  |
|  | 11. | | Dissatisfied with your sex life | | | | | | 0 | |  | 1 |  | 2 | 3 |  | 4 |  |
|  | 12. | | Angry about your sex life | | | |  |  | 0 | |  | 1 |  | 2 | 3 |  | 4 |  |
|  | 13. | | Bothered by low sexual desire | | | | | | 0 | |  | 1 |  | 2 | 3 |  | 4 |  |
