# Supplementary material for: Exploring the impact of urogenital organ displacement after abdominoperineal resection on urinary and sexual function
Source: Int J Colorectal Dis. 2022 Aug 31;37(10):2125–36. doi: 10.1007/s00384-022-04234-3 (PMC9562368; doi:10.1007/s00384-022-04234-3)
Supplement: Supplementary file 11 — Supplementary file11 (DOCX 20 KB) [file 384_2022_4234_MOESM11_ESM.docx]

**Supplementary Table 6** Baseline characteristics – compared with patients who did not return questionnaires

|  |  | **Questionnaires returned** | | **Questionnaires not returned** | |
| --- | --- | --- | --- | --- | --- |
|  |  | **Male total (n=89)** | **Female total (n=36)** | **Male total (n=85)** | **Female total (n=38)** |
| Age | Years (mean ± SD) | 63 ± 12 | 63 ± 14 | 66 ± 13 | 64 ± 12 |
| BMI | Kg/m^2^ (mean ± SD) | 27 ± 4 | 27 ± 5 | 27 ± 6 | 27 ± 7 |
| Prior pelvic surgery | Total | 7/89 (8) | 13/36 (36) | 22/85 (26) | 16/38 (42) |
|  | Female reproductive system^#^ | - | 10/36 (28) | - | 14/38 (37) |
|  | Male reproductive system^&^ | 1/89 (1) | - | 8/85 (9) | - |
|  | Lower urinary tract^+^ | 1/89 (1) | 0/36 (0) | 4/85 (5) | 0/8 (0) |
|  | Gastro intestinal~ | 5/89 (6) | 3/36 (8) | 14/85 (17) | 3/38 (8) |
| APR indication | Primary rectal cancer | 86/89 (97) | 35/36 (97) | 71/85 (84) | 31/38 (82) |
|  | Recurrent rectal cancer | 3/89 (3) | 1/36 (3) | 14/85 (16) | 7/38 (18) |
| Neo-adjuvant treatment | None | 9/89 (10) | 2/36 (6) | 14/83 (17) | 6/38 (16) |
|  | Short-course radiotherapy | 13/89 (15) | 8/36 (22) | 12/83 (15) | 4/38 (11) |
|  | Long-course radiotherapy | 14/89 (16) | 11/36 (31) | 24/83 (29) | 8/38 (21) |
|  | Chemoradiotherapy | 53/89 (60) | 15/36 (42) | 33/83 (40) | 20/38 (53) |
| APR type | Intersphincteric | 6/88 (7) | 0/36 (0) | 16/82 (20) | 4/38 (11) |
|  | Conventional | 14/88 (16) | 8/36 (22) | 27/82 (33) | 7/38 (18) |
|  | Extralevator | 68/88 (77) | 28/36 (78) | 39/82 (48) | 27/38 (71) |
| Multivisceral resection | Total | 32/89 (36) | 17/36 (47) | 25/82 (31) | 22/38 (58) |
|  | Vaginal wall | - | 9/36 (25) | - | 16/38 (42) |
|  | Adnex | - | 3/36 (8) | - | 5/38 (13) |
|  | Uterus^@^ | - | 2/30 (7) | - | 4/38 (11) |
|  | Seminal vesicle | 6/89 (7) | - | 4/82 (5) | - |
|  | (Partial) Prostate | 8/89 (9) | - | 9/82 (11) | - |
|  | (Partial) Bladder | 1/89 (1) | 0/36 (0) | 2/82 (2) | 0/38 (0) |
|  | Coccyx | 18/89 (20) | 3/36 (8) | 11/82 (13) | 7/38 (18) |
|  | Pelvic side wall | 2/89 (2) | 2/36 (6) | 3/82 (4) | 1/38 (3) |
|  | Presacral fascia | 0/89 (0) | 1/36 (3) | 1/82 (1) | 0/38 (0) |
| Omentoplasty | Total | 43/89 (48) | 17/36 (47) | 64/85 (75) | 20/38 (53) |
| Retroflexion uterus^+^ | Total | - | 1/18 (6) | - | 4/38 (11) |
| Perineal closure | Primary closure | 29/89 (33) | 14/36 (39) | 62/82 (76) | 24/38 (63) |
|  | Biological mesh | 44/89 (49) | 15/36 (42) | 4/82 (5) | 4/38 (11) |
|  | Resorbable synthetic mesh | 13/89 (15) | 4/36 (11) | 15/82 (18) | 5/38 (13) |
|  | Gluteal turnover flap | 2/89 (2) | 1/36 (3) | 0/82 (0) | 1/38 (3) |
|  | Muscle flap | 1/89 (1) | 2/36 (6) | 1/82 (1) | 4/38 (11) |

Data are presented as absolute numbers (proportions), unless otherwise stated
BMI: Body Mass Index; SD: Standard Deviation; TURP: Transurethral Resection of the Prostate; TURBT: Transurethral Resection of Bladder Tumour;

TEM: Transanal Endoscopic Microsurgery; APR: Abdominoperineal Resection
*more than one of the variables below might be applicable to the same patient

^#^Female reproductive system includes sacral colpopexy, sectio caesarea, oophorectomy, hysterectomy, sterilization, excision ovarial cyst
^&^Male reproductive system includes (partial) prostatectomy and TURP
^+^Lower urinary tract includes TURB
^~^Gastrointestinal includes TEM, LAR, perianal abscess
^@^patients with a prior hysterectomy were excluded
^+^patients with a prior hysterectomy or hysterectomy during operation were excluded
