# Supplementary material for: Exploring the impact of urogenital organ displacement after abdominoperineal resection on urinary and sexual function
Source: Int J Colorectal Dis. 2022 Aug 31;37(10):2125–36. doi: 10.1007/s00384-022-04234-3 (PMC9562368; doi:10.1007/s00384-022-04234-3)
Supplement: Supplementary file 12 — Supplementary file12 (DOCX 13 KB) [file 384_2022_4234_MOESM12_ESM.docx]

| **Supplementary Table 6.** Baseline urogenital function using The European Organization for Research and Treatment for Cancer Quality of Life Questionnaire Colorectal cancer (EORTC QLQ-CR29) | | | |  |
| --- | --- | --- | --- | --- |
| **Domain** | ♂ **Score (mean SD)** | ♀ **Score (mean SD)** |  |  |
| Urinary function |  |  |  |  |
| Urinary frequency | 32.1 ±24.9 | 22.2 ±27.6 |  |  |
| Urinary incontinence | 2.4 ±8.9 | 11.1 ±16.7 |  |  |
| Dysuria | 3.7 ±10.7 | 0.0 ±0.0 |  |  |
| Sexual function |  |  |  |  |
| Sexual interest | 71.6 ±28.8 | 91.7 ±15.4 |  |  |
| Impotence | 29.2 ±33.1 | Not applicable |  |  |
| Dyspareunia | Not applicable | 0.0 ±0.0 |  |  |
