# Supplementary material for: Exploring the impact of urogenital organ displacement after abdominoperineal resection on urinary and sexual function
Source: Int J Colorectal Dis. 2022 Aug 31;37(10):2125–36. doi: 10.1007/s00384-022-04234-3 (PMC9562368; doi:10.1007/s00384-022-04234-3)
Supplement: Supplementary file 13 — Supplementary file13 (DOCX 16 KB) [file 384_2022_4234_MOESM13_ESM.docx]

**Supplementary Table 7.** Spearman correlation between the angle rotation of the landmark and functional score

| **Gender** | **Landmark** | **Questionnaire** | | **Total** | | | **Sexually active** | | | **Sexually inactive** | | |
| --- | --- | --- | --- | --- | --- | --- | --- | --- | --- | --- | --- | --- |
|  |  |  | **Domain** | **n** | **ρ** | **P-value** | **n** | **ρ** | **P-value** | **n** | **ρ** | **P-value** |
| ♂ | Internal urethra orifice | UDI-6 |  | 82 | 0.088 | 0.433 |  |  |  |  |  |  |
| ♂ | Internal urethra orifice | UDI-6 | Obstructive | 83 | 0.070 | 0.529 |  |  |  |  |  |  |
| ♂ | Internal urethra orifice | IIQ-7 |  | 84 | 0.092 | 0.404 |  |  |  |  |  |  |
| ♀ | Internal urethra orifice | UDI-6 |  | 31 | -0.068 | 0.715 |  |  |  |  |  |  |
| ♀ | Internal urethra orifice | UDI-6 | Obstructive | 31 | -0.046 | 0.807 |  |  |  |  |  |  |
| ♀ | Internal urethra orifice | IIQ-7 |  | 30 | -0.017 | 0.931 |  |  | Not applicable | |  |  |
| ♂ | Posterior bladder wall | UDI-6 |  | 82 | 0.040 | 0.719 |  |  |  |  |  |  |
| ♂ | Posterior bladder wall | UDI-6 | Obstructive | 83 | -0.011 | 0.920 |  |  |  |  |  |  |
| ♂ | Posterior bladder wall | IIQ-7 |  | 84 | 0.039 | 0.723 |  |  |  |  |  |  |
| ♀ | Posterior bladder wall | UDI-6 |  | 33 | -0.163 | 0.364 |  |  |  |  |  |  |
| ♀ | Posterior bladder wall | UDI-6 | Obstructive | 33 | -0.269 | 0.130 |  |  |  |  |  |  |
| ♀ | Posterior bladder wall | IIQ-7 |  | 33 | -0.184 | 0.304 |  |  |  |  |  |  |
| ♂ | Distal end of prostatic urethra | IIEF |  | 63 | 0.014 | 0.913 | 36 | -0.086 | 0.618 | 27 | 0.261 | 0.189 |
| ♀ | Cervix/top of vagina | FSFI |  | 19 | 0.172 | 0.482 | 12 | 0.301 | 0.342 | 7 | 0.393 | 0.383 |
| ♀ | Cervix/top of vagina | FSFI | Pain | n.a. | n.a. | n.a. | 12 | 0.250 | 0.434 | n.a. | n.a. | n.a. |
| ♀ | Cervix/top of vagina | FSDS-R |  | 25 | 0.110 | 0.601 | 15 | 0.406 | 0.133 | 9 | -0.392 | 0.297 |

UDI-6 = Urogenital Distress Inventory short form; IIQ-7 = Incontinence Impact Questionnaire short form; IIEF = International Index of Erectile Function; FSFI = Female Sexual Function Index (converted); FSDS-R = Female Sexual Distress Scale – Revised
